# Topology entropy: Enhancing graph partitioning for TAD identification and single-cell clustering

**DOI:** 10.1016/j.csbj.2025.04.037

**Published:** 2025-04-30

**Authors:** Qiushi Liang, Shengjie Zhao, Lingxi Chen, Shuai Cheng Li

**Affiliations:** a School of Computer Science and Technology, Tongji University, Shanghai, 201804, China; b Department of Computer Science, City University of Hong Kong, 999077, Hong Kong, China; c Department of Biomedical Sciences, City University of Hong Kong, 999077, Hong Kong, China; d Engineering Research Center of Key Software Technologies for Smart City Perception and Planning, Ministry of Education, China

**Keywords:** Graph partitioning, Topology entropy, TAD identification, Hi-C contact map, Cell clustering, Single cell

## Abstract

Entropy quantifies the limits of information compression and provides a theoretical foundation for exploring complex structures in large-scale graphs. However, effective metrics are needed to capture the intricate structural details in biological graphs. In this paper, we introduce the *topology entropy encoding tree* to quantify the complexity of biological graphs and show that minimizing the associated entropy is equivalent to optimal graph partitioning. We develop two methods, TEC-O and TEC-U, for partitioning ordered and unordered biological graphs. TEC-O is applied to identify Topologically Associated Domains (TADs) in Hi-C contact maps, while TEC-U is used for cell clustering in single-cell sequencing data. Results from simulated datasets demonstrate that topology entropy is robust to noise and effectively captures structural information, outperforming existing methods. Experiments on Hi-C data from five cell lines and ten single-cell sequencing datasets show that TEC-O and TEC-U achieve the highest accuracy in TAD detection and cell clustering, respectively, providing biologically meaningful insights.

## Graphical abstract

**Figure:**
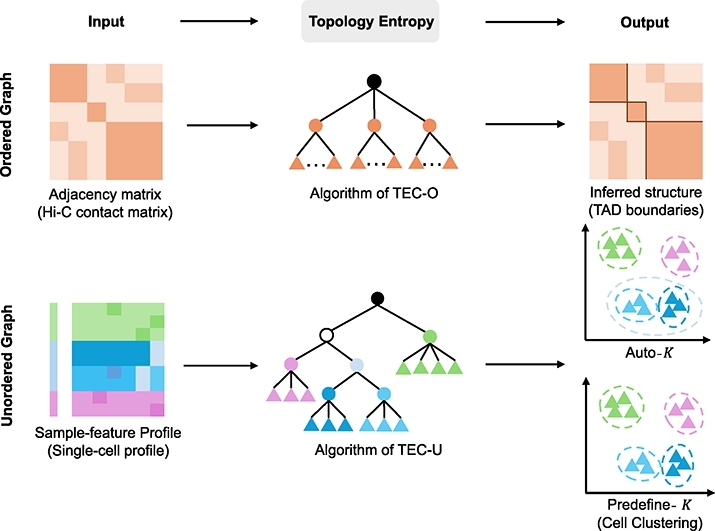

## 1 Introduction

Entropy serves as a fundamental measure for quantifying the limits of information compression and provides a theoretical basis for exploring complex structures in large-scale graphs, applicable to both ordered and unordered graphs. An *ordered graph* is a symmetric graph where vertices are arranged in a specific linear order. The partitions of an ordered graph consist of consecutive vertices defined by this vertex ordering. In contrast, an *unordered graph* represents a more general structure that allows for arbitrary arrangements of vertices.

High-throughput Chromosome Conformation Capture (Hi-C) contact maps naturally form a genome-wide ordered graph of genomic regions. This characteristic enables the application of graph partitioning methods to identify topologically associated domains (TADs) from Hi-C contact matrices. The Hi-C technique [1] captures the ligated fragments and identifies the chromatin interactions. The pipeline presented in [2] transforms raw sequencing reads into a Hi-C contact map graph. As the fundamental units of the genome, TADs consist of continuous bins that interact more closely with each other than with bins from different TADs [3]. TADs are closely related to epigenetic and transcriptional activities [4], and variations in TAD boundaries can result in cancers and developmental disorders [5]. Therefore, accurate identification of TADs in Hi-C matrices warrants thorough exploration.

Recent advances have explored entropy-based graph partitioning strategies for TAD identification. Li et al. [6] developed a structural entropy-based method to identify structures with minimal perturbations caused by random variations and noise. They subsequently derived a greedy algorithm to identify TADs with minimal structural entropy through iterative merging and combining [7]. Norton et al. [8] applied a Louvain-like, locally greedy approach to TAD identification by optimizing network modularity using a single resolution parameter. Considering the order of bins, the Hi-C contact map can be represented as an ordered graph, allowing the use of dynamic programming (DP) method can be used to achieve the global optimal solution. Zhang et al. [9] designed a DP method to search for the encoding tree of the contact map graph with minimal structural entropy. Li et al. [10] developed deDoc2 to seek genome partitions with globally minimal structural entropy for both whole and local contact matrices. The success of structural entropy and DP in TAD identification motivates our exploration of new entropy metrics.

The functional diversity of cells results from genetic variation, biological evolution, and the differentiation of cellular structure and function. Constructing a cell hierarchy can help decipher the functional diversity. For instance, infiltrated lymphocytes in the tumor microenvironment are classified into T cells and B cells, with T cells further divided into cytotoxic T cells and helper T cells [11]. Recent advancements in single-cell sequencing technologies have provided opportunities for large-scale analyses in transcriptomics [12], genomics [13], and epigenomics [14]. These technologies have led to revolutionary insights into cellular heterogeneity and complexity, enabling the grouping of cells with similar molecular traits into the same category [15]. By performing clustering at the single-cell level, we can identify not only distinct cell subpopulations that provide broad categories, such as T cells and B cells, but also more specific groupings within these subpopulations, such as cytotoxic T cells and helper T cells.

Various methods have been employed for single-cell clustering, including Louvain [16], Leiden [17], Infomap [18], K-Means [19], Hierarchical Clustering [20], and Spectral Clustering [21]. Louvain and Leiden optimize modularity to identify clusters, while Infomap is an entropy-based method grounded in the map equation. Infomap can be sensitive to noise within the graph, potentially leading to incorrect community assignments in noisy graphs [22]. K-Means is a centroid-based partitioning algorithm that iteratively assigns data points to clusters by minimizing the within-cluster sum of squared distances. Hierarchical clustering constructs a tree-like dendrogram to represent nested clusters, while spectral clustering transforms data into a similarity graph, applying eigenvalue decomposition on the graph Laplacian for partitioning. These methods have been utilized in various studies [23], [24], [25], [26], [27]. Despite their widespread application, research on entropy-based methods for single-cell clustering remains insufficient. The aforementioned approaches either require a predefined number of clusters *K* or can determine the value of *K* only after computation, highlighting the need for a method that can simultaneously provide both functionalities.

In this study, we propose a novel metric named *topology entropy* to quantify the complexity of graphs, based on the concept of mutual information. We construct a *topology entropy encoding tree* to facilitate graph partitioning and develop Topology Entropy-based Clustering (TEC) methods for both ordered and unordered biological graphs. For ordered graphs, we present an optimal algorithm, TEC-O, which utilizes DP and operates in polynomial time. In contrast, for unordered graphs, we propose a heuristic algorithm, TEC-U, that constructs a topology entropy encoding tree in an agglomerative manner and employs DP to contract the hierarchical tree, yielding the corresponding partitioning results. Furthermore, we offer two modes for TEC-U: predefined-*K* and auto-*K*, depending on whether the value of *K* is predetermined. We validate our approaches utilizing both simulated and real datasets, including various resolution Hi-C contact matrices for GM12878, IMR90, K562, NHEK, and HMEC cell lines, high-resolution Micro-C interaction data, eight single-cell RNA sequencing (scRNA-seq) datasets, and two single-cell assay for transposase-accessible chromatin (scATAC) datasets incorporating gold standard cell type labels. Experiments demonstrate that topology entropy effectively encodes structural information, and our methods consistently outperform other approaches. Our source code is available at https://github.com/keyqing5/TEC.

## 2 Methods

**Fig. 1:**
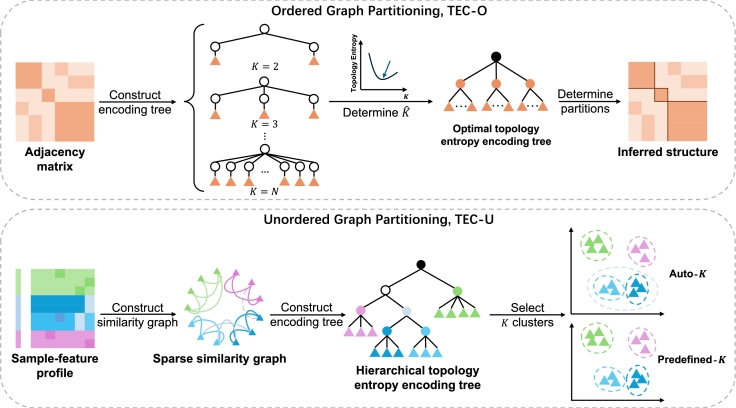
Workflow of clustering algorithms for ordered and unordered graphs. We developed distinct algorithms tailored for both ordered and unordered graphs. For ordered graph partitioning, the input is the adjacency matrix of the graph. Our TEC-O algorithm employs DP to construct a topology entropy encoding tree, which facilitates the identification of the optimal value of *K*. Conversely, for unordered graphs, the input is the sample-feature profile. Our TEC-U algorithm heuristically constructs a hierarchical encoding tree, followed by a contraction step for pruning. The predefined-*K* mode yields results based on a specified *K*, while the auto-*K* mode determines the optimal *K* and corresponding partitions automatically.

In this section, we first introduce the theoretical foundation of our graph partitioning algorithms, including the construction of an encoding tree and the computation of the tree nodes within the encoding tree. We then define the notion of topology entropy and identify the optimal topology entropy encoding tree. For ordered graphs, we present TEC-O, a DP approach to find the optimal topology entropy encoding tree. In contrast, for more general unordered graphs, we propose a heuristic method, TEC-U, which constructs the topology entropy encoding tree in an agglomerative manner and employs DP to contract the hierarchical tree, yielding the corresponding partitioning results. Additionally, depending on whether the value of *K* needs to be predefined, we offer two modes: predefined-*K* and auto-*K*. Through TEC-O and TEC-U, we introduce a new metric to measure the dynamics and randomness of graphs, facilitating a comprehensive exploration of graph partitioning techniques. The overall workflow of our study is depicted in Fig. 1.

### 2.1 Definitions

#### 2.1.1 Encoding tree

Let G=(V,E,w) be a non-negative weighted graph, where *V* and *E* are the set of vertices and edges, respectively, |V|=n, and $w:E\to R_{\geq 0}$. In this work, we assume the graphs are undirected. Let $w_{uv}$ represent the weight of the edge connecting vertices *u* and *v* in *G*.

An *encoding tree T* of *G* indicates the hierarchical partitioning of the vertex set *V*. The root *r* of *T* represents, or encodes, the entire vertex set *V*. For notation simplicity, we denote graph vertices by *u* and *v*, and tree nodes by *μ* and *ν*. Each tree node *μ* encodes a vertex subset V(μ), V(μ)⊂V. The children of each tree node partitions the vertices encoded by their parent node. For each vertex *u*, there exists a unique leaf node t(u)∈T such that u∈V(t(u)). Each leaf node encodes one or more vertices. An ideal encoding tree is hierarchical, where each tree node represents a partition with a finer resolution than that of its parent node. The vertices encoded by each tree node should exhibit a high degree of similarity.

Denote the volume of *μ* as the sum of weights of all vertices in V(μ), $vol(\mu )=\sum_{u\in V(\mu ),v\in V}w_{uv}$. For the volume of root *r*, we have $vol(r)=vol(G)=\sum_{u,v\in V}w_{uv}$. This indicates that the volume of *r* is equivalent to the volume of the entire graph *G*, which is defined as the sum of the weights of all edges in the edge set *E*. Define g(μ) as the total weights of edges from vertices in V(μ) to V−V(μ), $g(\mu )=\sum_{u\in V(\mu ),v\in V-V(\mu )}w_{uv}$. Suppose s(μ) is the total weights of edges between vertices in V(μ), $s(\mu )=\sum_{u\in V(\mu ),v\in V(\mu )}w_{uv}$. We have vol(μ)=g(μ)+s(μ). Clearly, if a tree node *μ* encodes one vertex, g(μ)=vol(μ).

#### 2.1.2 Topology entropy

Prior to defining our notion, it is worth recalling the modularity score [28] for a graph. Based on the formulation of the encoding tree, the definition of modularity can be rewritten as

(1) $$M_{T}(G;\mu )=\frac{s(\mu )}{vol(G)}-\gamma {\left(\frac{vol(\mu )}{vol(G)}\right)}^{2},$$

where *γ* is a resolution parameter, finer resolution leads to more partitions, while coarser resolution leads to fewer partitions (the specific rewriting process is detailed in Appendix A).

Clearly, the first term in Eq. (1) can be interpreted as a joint distribution $P_{\mu }$ while the second term represents the marginal distribution $Q_{\mu }$, that is,

(2) $$P_{\mu }=\frac{s(\mu )}{vol(G)},Q_{\mu }=\gamma {\left(\frac{vol(\mu )}{vol(G)}\right)}^{2}$$

In this work, we define *topology entropy* or *topology divergence*, which utilize Kullback-Leibler (KL) divergence to measure the difference between joint distribution *P* and marginal distribution *Q*. The KL divergence is formulated as

(3) $$D(P||Q)=\sum_{\mu }P_{\mu }\log\frac{P_{\mu }}{Q_{\mu }}.$$

We substitute $P_{\mu }$ and $Q_{\mu }$ from Eq. (2) into Eq. (3) and take the negative of the entire equation. To address the issue of the limited scale of communities, we introduce parameters $\alpha_{1}$ and $\alpha_{2}$ into the definition, similar to *γ* in Eq. (1), resulting in the following formulation:

(4) $$D_{T}(G;\mu )=-{\left(\frac{s(\mu )}{vol(G)}\right)}^{\alpha_{1}}\left(\log\frac{s(\mu )}{vol(G)}-\alpha_{2}\log\frac{vol(\mu )}{vol(G)}\right),$$

where $\alpha_{1}$ and $\alpha_{2}$ are resolution parameters that enable us to alter the relative weight given to the observed and randomized edge terms (the specific deviation process of Eq. (4) is detailed in Appendix B). The topology entropy of the root node *r* is 0, i.e., $D_{T}(G;r)=0$. With larger $\alpha_{1}$ and $\alpha_{2}$, the method tends to identify larger communities. With smaller $\alpha_{1}$ and $\alpha_{2}$, the method tends to identify smaller communities.

Let $D_{T}(G)$ denote the topology entropy of the entire encoding tree. The value of $D_{T}(G)$ is calculated as the sum of the topology entropy of all nodes in *T*, i.e., $D_{T}(G)=\sum_{\mu \in T}D_{T}(G;\mu )$. The definition of topology entropy can be interpreted as the negative value of the KL divergence between P and Q. In Eq. (3), we need to maximize D(P||Q) to obtain the optimal result. Therefore, our objective is to minimize the value of topology entropy, thereby reducing the global variance of the random walk on *G*. Finding the optimal topology entropy encoding tree can be further reformulated as finding a tree $T_{\text{opt}}$ with the minimum topology entropy for graph *G*, that is

(5) $$D_{T_{\text{opt}}}(G)=\underset{T}{\min}\;\sum_{\mu \in T}D_{T}(G;\mu ).$$

Based on the above, we transform the graph partitioning problem into the task of finding the optimal encoding tree with respect to topological entropy.

### 2.2 Finding the optimal solution on ordered graph

For ordered graph *G*, we can obtain the optimal solution using DP. Let D(i,k) represent the topology entropy of the optimal encoding tree that partitions vertices {1,...,i} with *k* disjoint parts. Additionally, let H(i:j) denote the topology entropy of the tree node encoding vertices {i,...,j}, 1≤i≤j≤n. We define the recursive function to determine the optimal encoding tree for vertices $\left\{1,...,i_{r}\right\}$ with *k* leaves as

(6) $$D(i_{r},k)=\underset{i\in \{1,...,i_{r}-1\}}{\min}\;\left(D(i,k-1)+H(i+1:i_{r})\right),$$

where

(7) $$H(i_{l}:i_{r})={\left(\frac{s(i_{l}:i_{r})}{vol(G)}\right)}^{\alpha_{1}}\left(\log\frac{s(i_{l}:i_{r})}{vol(G)}-\alpha_{2}\log\frac{vol(i_{l}:i_{r})}{vol(G)}\right),$$

and

(8) D(i,1)=H(1:i).

When calculating each value in *D*, the corresponding value of *i* is stored. During the backtracing process, we start from k=n−1 and retrieve the optimal value of *i* for partitioning the vertices. The backtrace continues until k=1, at which node further partitioning of the vertices is no longer possible. If there is a need to limit the maximum number of partitions to *K*, the backtracing process can start from D(n,K). In the final partition results, cases where k=1 or k=n imply that no partitioning has occurred. Therefore, we exclude these cases when evaluating the optimal partition results.

There are $O(n^{2})$ possible values for H(i+1;n), each of which can be computed in O(1) time. Therefore, calculation of *H* takes time $O(n^{2})$. The size of the table storing D(n,k) is O(kn). Computing each value of D(n,k) needs O(nk) time. Consequently, the time complexity of TEC-O of *k* leaves is $O(n^{2})$.

The above discussion addresses a single layer of partitioning. To establish a hierarchical structure, we iteratively compute optimal partitions layer by layer using Eq. (6). Specifically, after obtaining the partitioning at a higher level, the current adjacency matrix is divided into multiple sub-matrices based on the identified boundaries. We then apply the same partitioning procedure to each sub-matrix to uncover the sub-partitioning structure. Importantly, when a TAD consists of only a few bins, we refrain from performing any further partitioning.

### 2.3 Finding the hierarchical solution on unordered graph

In this section, we consider extending our graph partitioning method from ordered graphs to unordered graphs. The input is converted into a more general sample-feature matrix. We aim to develop a graph partitioning (or clustering) algorithm that takes the sample-feature matrix as input and identifies the optimal topology entropy encoding tree. The algorithm could have significant applications in various problems, including constructing cell hierarchies. While clustering algorithms such as K-means [19] and Hierarchical Clustering [20] demand the exact value of *K* to be predefined before computation, algorithms such as Louvain [16] and Leiden [17] do not require *K* to be specified in advance; instead they reveal *K* value only after the clustering results are finalized. To address this limitation, TEC-U offers two optional modes: it can perform clustering with a predefined *K* or heuristically search for the optimal *K*. For convenience, we refer to these two modes as *predefined-K* mode and *auto-K* mode, respectively.

The input sample-feature profile is reduced dimensionality to form a matrix *X*, followed by the construction of a corresponding dense sample-sample similarity graph G=(V,E). The dense graph is constructed using a Gaussian kernel $e_{u,v}=\text{exp}\left(-\frac{||x_{u}-x_{v}|{|}^{2}}{2\sigma^{2}}\right)$, where *σ* represents the standard deviation of *X* and $e_{u,v}$ quantifies the similarity between two samples. If the input is a low-dimensional matrix, further reduction is unnecessary, and we can move directly to the next step of processing. From the dense graph, a sparse graph $G_{s}$ is created using the *k*-nearest neighbors (kNNs) method, assigning binary edge weight. For nodes *u* and *v*, if either node is among the top *k* nearest neighbors of the other in the dense graph, the weight of the edge connecting them in the sparse graph is set to 1; otherwise, the weight is set to 0. Our clustering algorithm leverages both the sparse and dense graphs to perform calculations, ultimately identifying the optimal encoding tree $T_{\text{opt}}(G)$ with minimal total topology entropy. There are two primary approaches for tree construction: top-down and bottom-up. In the design of TEC-U, we chose an agglomerative approach due to its potential for improved local merges, which helps to avoid premature erroneous decisions.

**Fig. 2:**
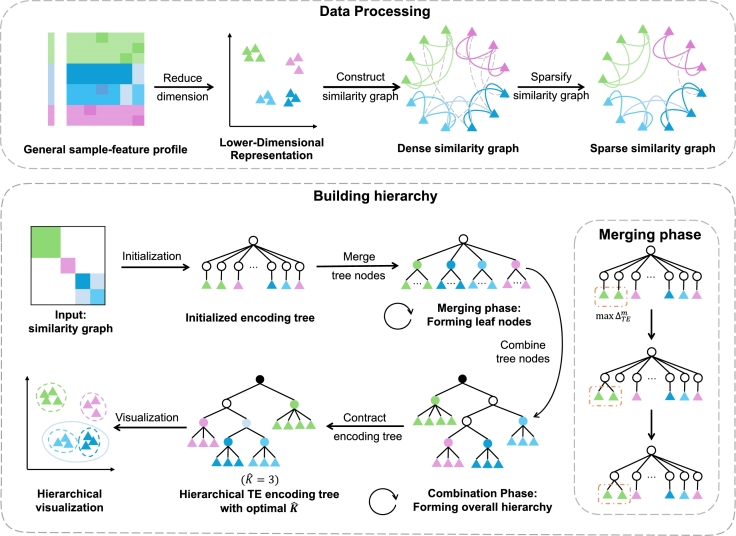
Workflow of TEC-U clustering algorithm on unordered graph.

Our clustering algorithm consists of four steps: initialization, merging phase, combination phase, and finding the optimal *K* for the hierarchical topology entropy encoding tree. In the initialization step, we add a root node *r* to the tree *T*, which has |V|=n direct leaf nodes. Each leaf node *t* encodes a distinct vertex *u* from the graph $G_{s}$, such that V(t)={u}. The processing of the input and the details of each step are depicted in Fig. 2. Subsequently, we will describe each step thoroughly in the order presented.

The merging phase consists of two nested iteration processes. Before detailing the iteration process, we need to explain the operation and properties of the tree nodes involved. We define two tree nodes, *μ* and *ν*, as *connected* when there exists at least one connecting edge between the vertices V(μ) and V(ν) in the encoded graph $G_{s}$. For a vertex u∈V(μ), we remove it from its current leaf node t(u)=μ and instead assign it to a different leaf node t(v)=ν, which is connected to *μ*. This results in new leaf nodes $\mu^{\prime }$ and $\nu^{\prime }$, where $\left\{u\}\subset V(\nu^{\prime }\right)$ and $\left\{u\}⊄V(\mu^{\prime }\right)$. We refer to this transformation as a *merging* operation. The change in the topology entropy value of the tree *T* before and after the merging can be computed as

(9) $$\Delta_{TE}^{m}(u,\mu ,\nu )=D_{T^{\prime }}(G_{s};\mu^{\prime })+D_{T^{\prime }}(G_{s};\nu^{\prime })-D_{T}(G_{s};\mu )-D_{T}(G_{s};\nu ).$$

If *μ* only encodes vertex *u*, then $D_{T^{\prime }}(G_{s};\mu^{\prime })=0$.

To facilitate discussion, we describe the two iterative processes as outer iteration and inner iteration. The inner iteration is used to perform merging at a more fine-grained resolution. In the inner iteration, for each vertex *u* in graph *G*, we compute the $\Delta_{TE}^{m}(u,\mu ,\nu )$ values for all possible candidates *ν* for merging resulting in a set of triples $\left(u,\nu ,\Delta_{TE}^{m}(u,\mu ,\nu )\right)$. We identify $\min(\Delta_{TE}^{m}(u,\mu ,\nu ))$ from the set of all triples, which indicates the vertex *u* and candidate *ν*. If $\min(\Delta_{TE}^{m}(u,\mu ,\nu ))<0$, we proceed with the corresponding merging and continue iterating; if $\min(\Delta_{TE}^{m}(u,\mu ,\nu ))\geq 0$, we stop the iteration. Thus, in each iteration, a leaf node can exist in one of two states: remaining unchanged or undergoing merging. When the inner iteration ends, we remove all leaf nodes that do not encode any vertices.

The outer iteration then recalculates the difference in topology entropy between the encoding tree $T^{\prime }$ after one round of inner iteration and the encoding tree *T* before the iteration, denoted as $\Delta D_{T}$. If $|\Delta D_{T}|\geq \Delta D_{thres}$, we merge all vertices associated with the same leaf node in $T^{\prime }$ into a single vertex, resulting in a new graph $G_{s}^{\prime }$, and then start a new round of inner iteration. Otherwise, the iteration stops, and the process moves on to the combination phase.

Additionally, we have set a bool parameter called *multis*, which indicates whether to perform the outer iteration. When the parameter *multis* is set to False, it allows the algorithm to run only the inner iteration, thereby completing the basic fine-grained vertex merging operations. The selection of *multis* does not require manual setting. Our algorithm can operate with both possible values, running once for each, and subsequently select the appropriate partitioning outcome based on the topology entropy value of the encoding trees. More details on the merging phase are mentioned in Appendix D.

Through the merging phase, we obtain an encoding tree *T* with *l* leaf nodes and one root node *r*. In the combination phase, we further aggregate the leaf nodes to achieve a coarser partitioning result, facilitating the subsequent selection of *K*. Here, we define the *combination* operation: for tree nodes *μ* and *ν*, we construct their parent node *ω*, which is a child node of the root *r*. The change in the encoding tree topology entropy before and after a combination can be computed as

(10) $$\Delta_{TE}^{c}(\mu ,\nu )=D_{T^{\prime }}(G_{s};\omega )+D_{T^{\prime }}(G_{s};\mu )+D_{T^{\prime }}(G_{s};\nu )-D_{T}(G_{s};\mu )-D_{T}(G_{s};\nu ).$$

In the combination phase, we first iteratively combine the connected tree nodes *μ* and *ν* that yield the smallest $\Delta_{TE}^{c}(\mu ,\nu )$ value. Then, we relax the constraints on connectivity and select the tree node pair that produces the smallest $\Delta_{TE}^{c}(\mu ,\nu )$ for combination. The combination iterates until the upper hierarchy forms a complete binary encoding tree.

After the combination phase, we obtain a hierarchical encoding tree with 2l−1 tree nodes. Next, we perform a pruning-like operation on tree *T* to automatically select the optimal *K* value that minimizes the topology entropy value $D_{T_{\text{opt}}}(G)$ for dense graph *G*. We equate the task of finding the optimal encoding tree to that of identifying an aggregation of optimal subtrees. Consequently, the optimal partitioning of the entire graph is decomposed into multiple subproblems. Specifically, when looking for *K* partitions of *G*, it can be formulated as finding two subtrees encoding $K^{\prime }$ and $K-K^{\prime }$ partitions, where $1\leq K^{\prime }<K$. Each tree node *ω* is treated as the root of a subtree $T_{\omega }$. We identify the subtree with the minimum topology entropy for $K\in \{1,2,\ldots ,K_{\text{max}}\}$ using DP, where $K_{\text{max}}$ is the maximum *K* value. For the subtree $T_{\omega }$, assuming *ω* has two children, *μ* and *ν*, we have

(11) $$J_{G}(\omega ,K)=\left\{ \begin{array}{cc} D(G;\omega ) & K=1,\\ \min_{K^{\prime }\in \{1,...,K-1\}}\{J_{G}(\mu ,K^{\prime })+J_{G}(\nu ,K-K^{\prime })\} & K\geq 2, \end{array}\right.$$

where D(G;ω) denotes the topology entropy of the subtree $T_{\omega }$ which effectively encodes a partition for vertices V(ω), and $J_{G}(\omega ,K)$ denotes the minimum topology entropy of the subtree $T_{\omega }$ with *K* children. Through the computation in Eq. (11), we generate a table that stores the values of $J_{G}(\omega ,K)$. Additionally, we create a table I(ω,K) storing the corresponding *μ* and *ν*, along with a cut-off table $K_{G,c}(\omega ,K)$ recording the corresponding $K^{\prime }$ for each minimum $J_{G}(\omega ,K)$ value obtained, i.e.,

(12) $$K_{G,c}(\omega ,K)=\arg\min_{K^{\prime }\in \{1,...,K-1\}}\{J_{G}(\mu ,K^{\prime })+J_{G}(\nu ,K-K^{\prime })\}.$$

During the backtrace process, we determine the optimal value of *K* as $\overset{ˆ}{K}$ by

(13) $$\overset{ˆ}{K}=\arg\min_{K\in \{1,...,K_{\text{max}}\}}J_{G}(r,K).$$

We then identify the children nodes *μ* and *ν* of the root based on table $I(\omega ,\overset{ˆ}{K})$ and $K_{G,c}(\omega ,\overset{ˆ}{K})$, continue to determine the children nodes of *μ* and *ν* until the corresponding value in $K_{G,c}$ equals 1. Through this backtrace, we derive the optimal topology entropy encoding tree $T_{\text{opt}}$, which corresponds to the minimum topology entropy value for $\overset{ˆ}{K}$ partitions. We then map the hierarchical structure of $T_{\text{opt}}$ to the partitioning results of the graph *G*. It is worth mentioning that in the encoding tree generated by TEC-U, the tree nodes representing the *K* clusters do not necessarily reside at the same layer; however, the vertices they encode do not overlap.

In practical applications, for larger input data, it is possible to appropriately reduce the setting of $K_{\text{max}}$ values to decrease the running time of computation.

For a sparse graph with *n* vertices, initialization requires O(n) time. The time complexity of the merging phase is O(nlog⁡n). In the combination phase, we use a heap to store the values of $\Delta_{TE}^{c}(\mu ,\nu )$, resulting in a time complexity of $O(l^{2}\log l)$, where l≪n. The process of selecting the optimal *K* using the DP tables has a time complexity of $O(lK_{\text{max}}^{2})$, where $K_{\text{max}}\ll n$. Therefore, the overall time complexity of the TEC-U algorithm is O(nlog⁡n).

### 2.4 Applying graph partitioning to TAD identification and single-cell clustering

A Hi-C contact map is equivalent to an ordered graph with its vertices arranged in a linear order. Each vertex in the graph indicates a bin (genomic region) in the contact map, and the edges represent the interaction frequencies between pairs of genomic bins. The linear order property allows us to employ efficient algorithms for further partitioning. Therefore, we apply the TEC-O algorithm described in Section 2.2 to the Hi-C contact matrix to identify the TADs.

Clustering analysis is routinely performed on single-cell sequencing data to explore and identify underlying cell identities. Constructing a cell hierarchy through cell clustering can help decipher the functional diversity of cells, which arises from genetic variation, biological evolution, and the differentiation of cellular structures and functions. Single-cell molecular data can be represented as a matrix, where the rows correspond to molecular features, such as genes or genomic regions, and the columns correspond to cells. Each matrix entry quantifies the value for a specific cell-feature pair, including measurements such as gene expression or copy number variation. Therefore, we input the cell molecular profiles and utilize the data processing procedure outlined in Section 2.3 to construct a cell-cell graph that captures the similarities between cells. This cell-cell graph is then applied to our algorithm, TEC-U, which facilitates cell clustering and represents the hierarchical relationships among the cells.

## 3 Results

### 3.1 Experiments on simulated ordered graph

Before conducting experiments with real data, we first validated the accuracy and robustness of TEC-O, using simulated ordered graph data. This allows us to assess the impact of different graph configurations on the results and compare TEC-O with other approaches.

We generated simulated Hi-C contact data incorporating different proportions of noise and TAD sizes. Specifically, the input is an adjacency matrix $X={\left\{x_{ij}\right\}}_{N\times N}$ representing a graph G=(V,E) with non-overlapping clusters, where $\forall x_{ij}\in \left\{0,1\right\}$. If edge $\left(v_{i},v_{j})\in E(G\right)$, then $x_{ij}=1$; otherwise, $x_{ij}=0$. The size (or length) of each TAD is indicated by the number of vertices contained within each cluster in the graph. We configured the vertices within each cluster to have a continuous adjacency relationship, ensuring a high probability of intra-interaction among the vertices to simulate the TADs observed in Hi-C contact data. Additionally, we established the presence of inter-interactions between different clusters to represent the noise in Hi-C contact data. The noise ratio is defined as the probability of inter-interaction relative to the probability of intra-interaction. A higher noise ratio indicates more edges between clusters, corresponding to increased noise. The number of TADs varies randomly between 8 and 10. We assigned a probability of 0.7 to the intra-connections within each cluster. Ground truth structures are obtained during generation. The simulation data are generated independently 100 times for each setting and input to all methods.

We compared the encoding trees corresponding to the inferred and ground truth TADs by calculating the values of weighted similarity [7] and overlapping ratio [9] (details in Appendix E). Both metrics evaluate the similarity between two encoding trees, with values ranging from 0 to 1. A value of 0 indicates no overlap between the two encoding trees, while a value of 1 indicates they are identical.

**Fig. 3:**
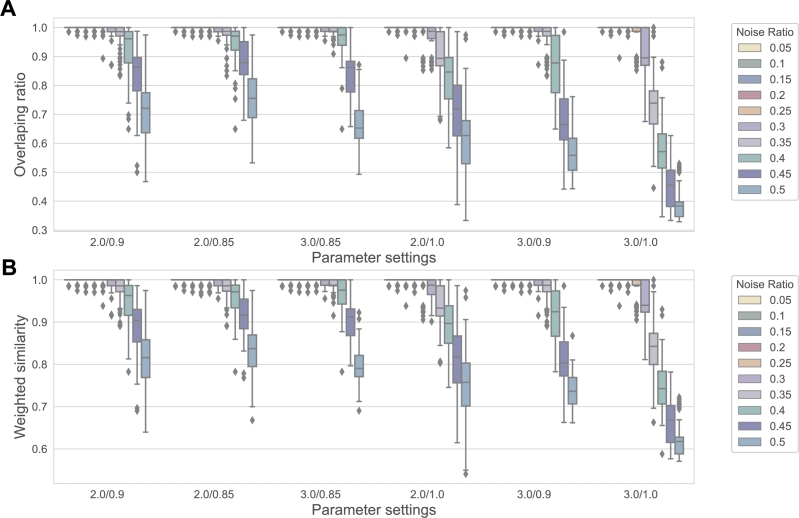
Comparison between different parameter settings (*α*_1_/*α*_2_) of topology entropy under various noise ratio. In the boxplot, the center line indicates the median of the metric, while the upper and lower boundaries of the box represent the third (Q3) and first (Q1) quartiles, respectively. The whiskers extend to 1.5 times the interquartile range (IQR, i.e., Q3 - Q1), and the points outside the whiskers represent outliers.

**Fig. J.13:**
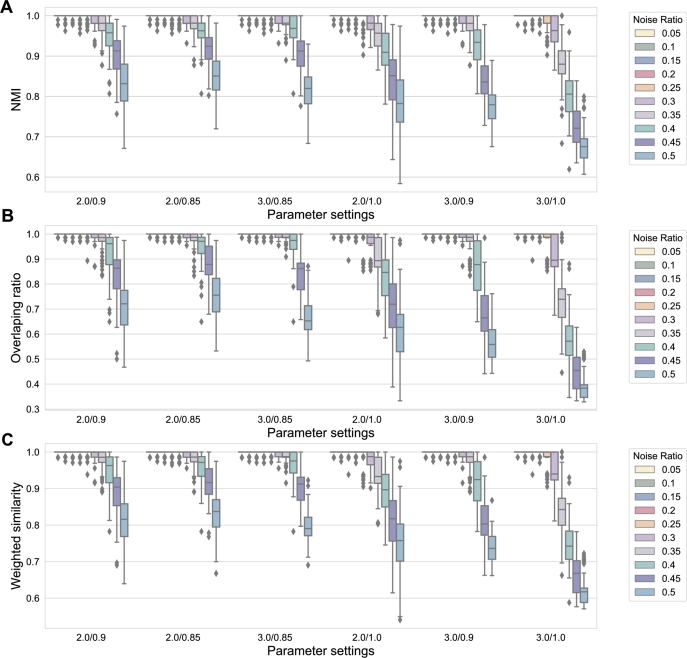
Comparison between different parameter settings (*α*_1_/*α*_2_) of topology entropy in the TEC-O algorithm under various noise ratios.

Before formally comparing with other baseline methods, we tested the impact of different values for parameters $\alpha_{1}$ and $\alpha_{2}$ in Eq. (4) on the experimental results. We set the deviation of TAD sizes to 3 and varied the noise ratio from 0.05 to 0.5. The combinations of $\alpha_{1}$ and $\alpha_{2}$ were set to (2.0, 0.9), (2.0, 0.85), (3.0, 0.85), (2.0, 1.0), (3.0, 0.9), and (3.0, 1.0). As shown in Fig. 3, the combination of $\alpha_{1}=2.0$ and $\alpha_{2}=0.85$ demonstrated the least sensitivity to noise in the data, leading to the best overall performance in terms of accuracy (more details in Fig. J.13). Therefore, we applied this parameter set in the subsequent experiments.

We compared results from SuperTAD [9], deDoc2 [10], and 3DNetMod [8]. These methods utilized structural entropy or modularity as their objective functions. Considering the performance of 3DNetMod in [9], we optimized it using DP to facilitate the comparison of different definitions of entropy. To validate the performance of our proposed topology entropy, we also replaced the topology entropy in TEC-O with modularity, denoted as TEC-O(M). All baseline methods were configured according to recommended or default parameters.

**Fig. 4:**
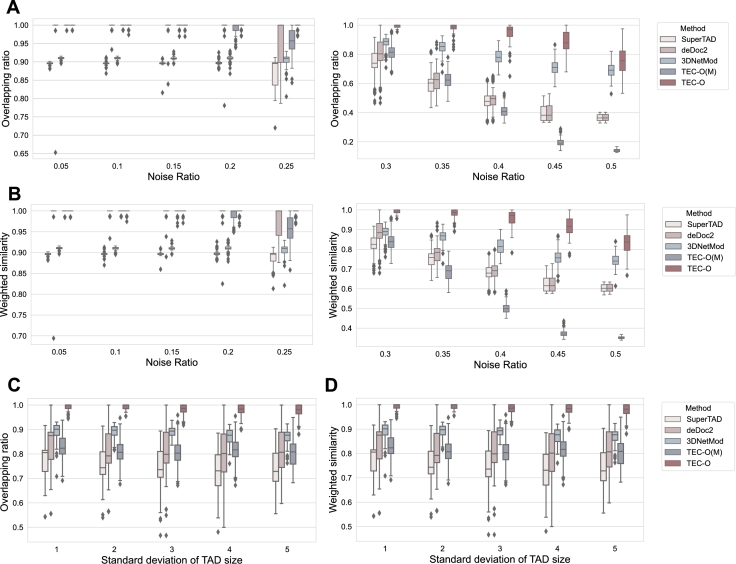
Robustness comparison under various noise ratios and deviations in TAD sizes. A-B. The x-axis denotes the noise ratio, increasing in 5% increments from 5% to 50%. C-D. The x-axis represents the deviation in TAD sizes, ranging from 1 to 5. The plots illustrate the distribution of specific metrics from 100 experiments under consistent parameter settings for input matrices. The box for TEC-O is colored dark red.

We evaluated the identification results of input matrices with varying noise ratios. The noise ratio was increased from 5% to 50% in increments of 5%, with the TAD size fixed at 10. As shown in Fig. 4A-B, when the noise ratio is less than or equal to 0.2, all methods achieve stable results with relatively high accuracy. However, as the noise increases, the accuracy significantly decreases, with notable changes in metrics at noise ratios of 0.25 and 0.3. When the noise ratio reaches 0.45 or higher, the performance of TEC-O(M) deteriorates significantly, with metric values approaching 0. At noise ratios of 0.4 and above, the results of deDoc2 and SuperTAD are similar, which we hypothesize is due to the inherent definition of entropy having a greater impact than differences in algorithm design. TEC-O, while showing a decrease in accuracy with increasing noise, maintains the highest average accuracy at a noise ratio of 0.5, with overlapping ratio and weighted similarity exceeding 0.7 and 0.8, respectively, outperforming other methods. Moreover, the accuracy of TEC-O is less affected by noise, demonstrating strong noise tolerance. The differences in metrics among the DP-based methods indicate that topology entropy can better assist in finding partitions close to the ground truth.

We also tested the effect of TAD size deviation on the performance of methods. With the noise ratio fixed at 0.3, we evaluated accuracy changes with TAD size deviations of {1,2,3,4,5}. As shown in Fig. 4C-D, larger deviations in TAD size lead to a decrease in accuracy, particularly noticeable between 1 and 2. The overlapping ratio and weighted similarity of TEC-O remain very close to 1, with only a slight decrease in accuracy, outperforming the other methods.

Overall, the results for overlapping ratio and weighted similarity indicate that variations in noise ratio and TAD size deviation significantly impact the accuracy of identification. TEC-O demonstrates higher accuracy and better robustness compared to other approaches.

Furthermore, we explored the sub-TAD structures and compared the performance. To facilitate direct comparisons, we have retained the names of the methods introduced previously without modification.

**Fig. F.11:**
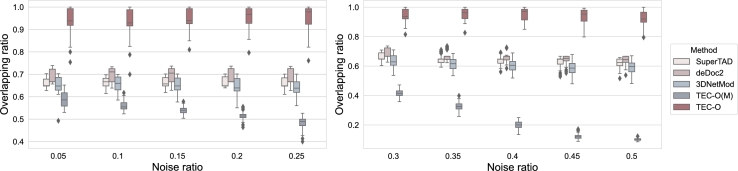
The robustness comparison under various noise ratios of input matrices with two-level sub-structures. The x-axis denotes the noise ratio, increasing in 5% increments from 5% to 50%. The plots illustrate the distribution of overlapping ratio from 100 experiments under the same parameter setting of matrices. The box for TEC-O is colored dark red.

For generating simulated data with two-level structures, we constructed TAD structures layer by layer and combined matrices from each layer. To facilitate comparison, we utilized the two-level results from baseline methods. We evaluated the identification results of the input matrices with varying noise ratios, increased from 5% to 50% in increments of 5%, while keeping the TAD size fixed at 10. As illustrated in Fig. F.11, TEC-O effectively partitioned the two-layer structures and demonstrated robustness against noise. In contrast, the TEC-O(M) method exhibited poor adaptability to the two-layer data and lacked robustness against noise. Notably, SuperTAD, deDoc2, and 3DNetMod showed comparable accuracy, with deDoc2 performing slightly better than the other two methods.

### 3.2 Real Hi-C contact data

We downloaded the processed real Hi-C contact dataset for chromosome 19 from the GM12878 and IMR90 cell lines at resolutions of 25 kb, 50 kb, and 100 kb, respectively [29]. The Hi-C contact matrices were derived from the normalized raw matrices using the Knight-Ruiz normalization method implemented in Juicer [2]. The start and end positions for the analysis were set at 7500000 bp and 22500000 bp, respectively. Additionally, we included datasets from K562, NHEK, and HMEC cell lines at a resolution of 25 kb, and conducted experiments with Micro-C data on human embryonic stem cells at resolutions of 5 kb and 10 kb. We aimed to evaluate the performance of methods across datasets with varying sequencing depths. Micro-C, as a high-resolution variant of Hi-C, enables a more detailed analysis of TADs. The Hi-C contact data for the cell lines were obtained from [29], while the Micro-C dataset was retrieved from [30] with accession number 4DNES21D8SP8, analyzed from positions 7500000 bp to 9300000 bp.

Furthermore, we generated subsamples of the contact data from the K562 cell line at different ratios (30%, 50%, and 70%) by randomly downsampling the pooled sample to further assess the robustness of methods. Specifically, downsampling was performed using FAN-C [31], resulting in normalized interaction matrices at a resolution of 25 kb.

### 3.3 Comparison of length and contact density of identified TADs

For the TADs identified in the real Hi-C contact matrices, we first calculated the TAD length (size) and contact density [7] distribution for each cell line using different methods. If the length of a TAD is too small, it may be filtered out in subsequent biological analyses. The contact density is calculated by dividing the sum of contacts within a TAD by the length of that TAD [7]. A high contact density of the identified TADs indicates that the quality of the TADs detected is high.

**Fig. 5:**
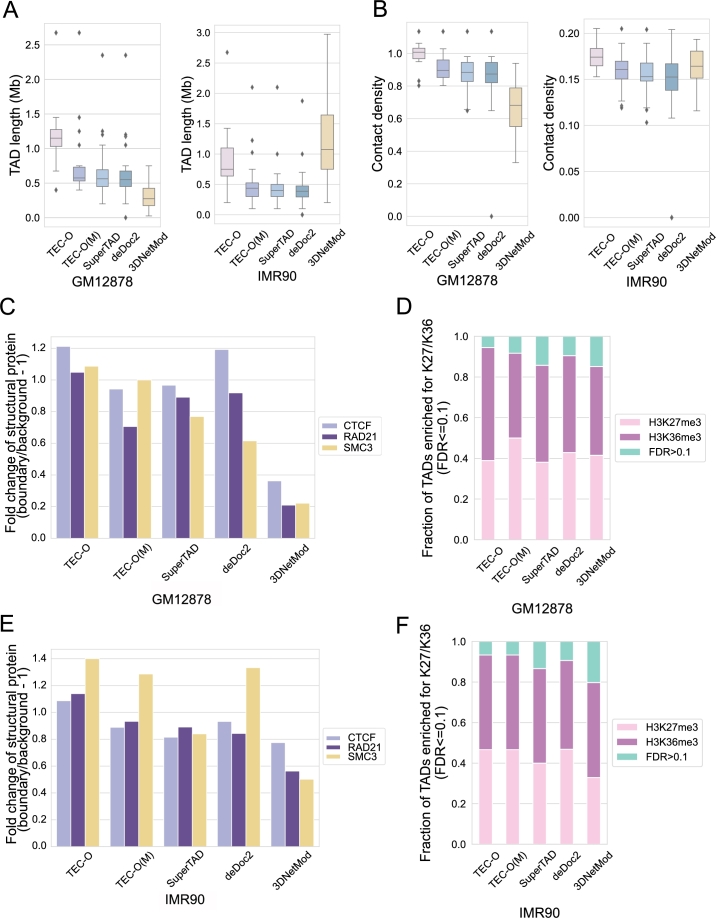
The TAD attribute statistics for all methods using a 25 kb resolution real Hi-C contact matrix on chromosome 19 for GM12878 and IMR90 cell lines. Box plots illustrate the inferred TADs regarding A. TAD length and B. contact density. Each boxplot illustrates the distribution of values for methods associated with a specific cell line. C and E. The fold change values of peak numbers of structural proteins (CTCF, RAD21, and SMC3) in peak regions compared to background regions. D and F. Cumulative bar charts displaying the proportions of TADs enriched for H3K27me3 or H3K36me3 (FDR corrected *p*-value ≤0.1), and those with no significant enrichment (i.e., FDR >0.1).

As illustrated in Fig. 5A, TEC-O detects TADs with longer lengths for the GM12878 cell line. The results obtained from TEC-O demonstrate that our entropy definition effectively prevents TADs from being excessively small, ensuring they are retained for analysis. For the GM12878 cell line, the lengths of TADs identified by 3DNetMod exhibit the smallest median value among all methods. Despite a wide range of lengths observed in 3DNetMod, with the largest TAD length for the IMR90 cell line, the contact density of the TADs identified by 3DNetMod, both in terms of maximum and median values, is lower than the corresponding values obtained using TEC-O. This observation suggests a potential instability in 3DNetMod. As shown in Fig. 5B, the median value of contact density of the TADs identified by TEC-O is the highest among all methods.

### 3.4 Enrichment of epigenetic characteristics at TAD boundaries or in TADs

The chromatin insulator protein CTCF and the cohesin complex, SMC3 and RAD21, are known to be enriched at TAD boundaries [29]. To assess this enrichment, we analyzed the binding of CTCF, SMC3, and RAD21 using ChIP-seq data. We quantified the enrichment by calculating the average fold change of peak enrichment between TAD boundaries and their flanking regions. The fold change was determined as the ratio of the number of peaks at TAD boundaries to those in the flanking regions, minus 1. Specifically, we calculated the number of peaks around TAD boundaries (±1 bin) and defined the flanking regions as 100 kb segments located 400 kb away from the boundaries. A high average fold change indicates significant protein enrichment at the boundaries, while a value of zero suggests no enrichment. We retrieved peak data for cells from the ENCODE (http://www.encodeproject.org).

As shown in Fig. 5C and E, boundaries identified by TEC-O exhibited higher fold changes for all structural proteins across both cell lines compared to other methods.

For GM12878 and IMR90 cell lines, we further evaluated the enrichment of histone modifications H3K27me3 and H3K36me3, following the analysis from [9], [32]. We computed the ratio of enrichment for either histone mark within each TAD by grouping ChIP-seq signals into intervals corresponding to 10% of the average TAD size. For each interval, we calculated the log10-ratio (LR) between H3K27me3 and H3K36me3. The average LR values were derived as the observed LR values, and the intervals were shuffled 1000 times to establish the distribution of LR values. We then calculated empirical *p*-values for TADs and adjusted the *p*-values for the false discovery rate (FDR) using the Benjamini-Hochberg procedure. We reported the ratio of enrichment with FDR-corrected *p*-values ≤0.1. A TAD was considered to show no enrichment for either histone mark if the FDR-corrected *p*-value exceeded 0.1. As illustrated in Fig. 5D and F, TEC-O identified a greater proportion of TADs with enriched histone modifications compared to other methods. Moreover, the proportion of TADs identified by TEC-O that were not enriched for either histone mark was the smallest among all methods. For more details, please refer to Table H.5, Table H.6, Table H.7, Table H.8.

For the K562, NHEK, and HMEC cell lines, as well as Micro-C contact data, we assessed the enrichment of regulatory elements, including CTCF, H3K27ac, H3K4me3, and H3K9me3. We found that CTCF, H3K4me3, and H3K27ac were enriched at TAD boundaries, while H3K9me3 was depleted. We evaluated the accuracy of the methods by calculating the fold change of these regulatory elements. The accession numbers are summarized in Table G.3, Table G.4.

As presented in Table I.9, Table I.10, TAD boundaries identified by TEC-O displayed enrichment across most regulatory elements, demonstrating the effectiveness of our algorithm and the utility of topology entropy in TAD identification.

As shown in Table I.11, the boundaries of TADs inferred from 100% of the total reads and those inferred from various ratios of downsampled reads became increasingly similar as sequencing depth increased for most callers. We quantified the similarity between TADs inferred from 100% of the reads and those from downsampled ratios using the overlapping ratio and weighted similarity metrics. The results from TEC-O demonstrate its robustness in TAD identification.

**Fig. F.12:**
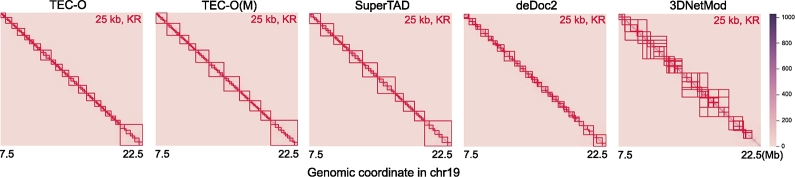
Heatmaps and the inferred two-level TAD boundaries of the Hi-C contact map for K562 cell line on chromosome 19 at 25 kb resolution.

For TAD structures with two levels, we tested the methods on the Hi-C contact matrix for the K562 cell line. As shown in Table F.2 and Fig. F.12, TADs identified by TEC-O exhibited high enrichment for CTCF, SMC3, and H3K4me3. Notably, during the two-layer analysis, the results from 3DNetMod were comparable to those from SuperTAD and deDoc2.

**Fig. 6:**
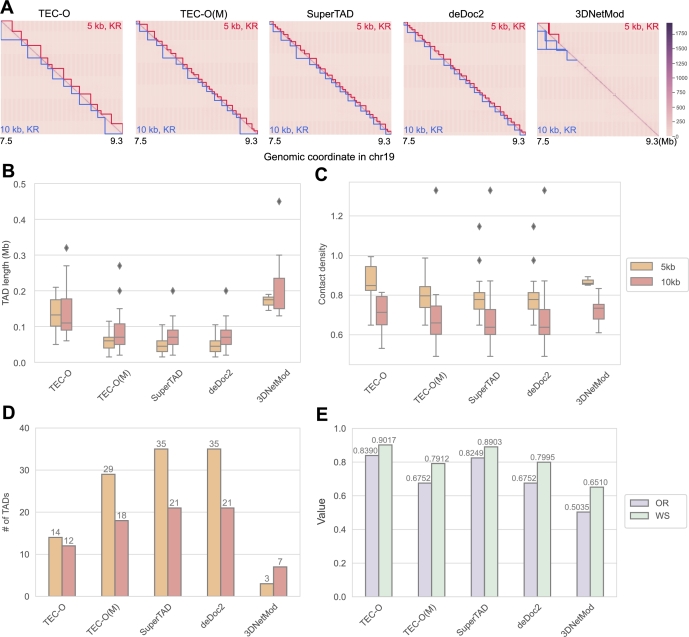
TAD identification on Micro-C data. A. Heatmaps of Micro-C contact data (chr19:7500000-9300000bp). Inferred TAD boundaries are annotated with resolution-specific colors (5 kb in red, 10 kb in blue). B-D. Boxplots and bar plots illustrate the inferred TADs regarding B. TAD length, C. contact density, and D. number of TADs. E. Consistency evaluation between 5 kb and 10 kb resolutions using overlapping ratio and weighted similarity.

For Micro-C contact matrices, as illustrated in Fig. 6, TEC-O identified TADs with greater average contact density. The results from deDoc2 and SuperTAD, both of which are based on structural entropy, were similar. After filtering, TADs identified by 3DNetMod were reduced to only 3 and 7 for the two resolutions. Regarding consistency across different resolutions, both TEC-O and SuperTAD produced robust results. Additionally, in Table I.12, TEC-O achieved higher fold change values for regulatory elements, including CTCF, H3K4me4, and RAD21, at both 5 kb and 10 kb resolutions.

### 3.5 Consistency between TAD boundaries at different resolutions

We evaluated the robustness and consistency of methods by comparing the similarity of TAD boundaries at different resolutions for cell lines. Specifically, we input Hi-C matrices with identical starting and ending sites on chromosome 19 at different resolutions into TEC-O and compare the similarity of TAD boundaries both qualitatively and quantitatively.

**Fig. 7:**
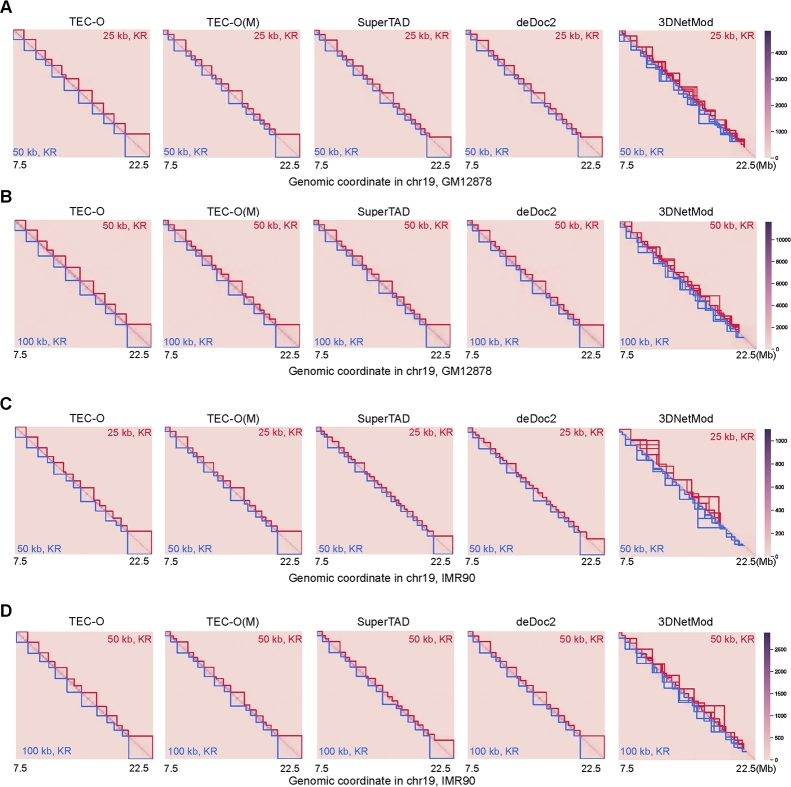
Consistency comparison for methods. We plotted the heatmap of the Hi-C contact matrix used as input and the inferred TAD boundaries for GM12878 (first two rows) and IMR90 (last two rows) cell lines. TAD boundaries are shown in the upper and lower triangles, with text within these triangles indicating the resolution and KR normalization of the corresponding input matrix. When comparing results across two resolutions, the domains calculated at a finer resolution are shown in red, while those at a coarser resolution are shown in blue. In each heatmap, the similarity between the boundaries reflects the consistency of the method across the matrices of these two resolutions.

The heatmaps of the input matrices for two cell lines, along with the identified TAD boundaries at 25 kb, 50 kb, and 100 kb resolutions, are depicted in Fig. 7. It is observable that when the input data resolution is finer, the methods tend to yield smaller domains. The differences among TEC-O, TEC-O(M), and SuperTAD in identifying TAD boundaries highlight the impact of the different definitions of entropy. 3DNetMod exhibits significant inconsistency in results across different resolutions due to excessive boundary duplications, leading to discontinuous filtered domains. In contrast, TEC-O demonstrates overall better consistency.

To quantify the consistency, we calculated the weighted similarity, normalized mutual information (NMI), and overlapping ratio for the TAD boundaries at different resolutions. As shown in Table 1, all methods exhibit a relatively high consistency in the comparisons between 25 kb and 50 kb for GM12878, as well as between 50 kb and 100 kb for IMR90. Due to the duplications in boundaries and the filtering settings, 3DNetMod consequently shows vast disagreement between results. Among all results, TEC-O demonstrates higher consistency in outputs for both GM12878 and IMR90 cell lines across different resolutions. TEC-O demonstrates higher consistency compared to TEC-O(M), further validating the robustness of our topology entropy approach and its applicability in addressing TAD detection challenges.

**Table 1:** Evaluation of similarity criteria between results at 25 kb, 50 kb, and 100 kb resolutions across all methods.

Cell Line		Metric	Method				
			TEC-O	TEC-O(M)	SuperTAD	deDoc2	3DNetMod
GM12878	25/50*	OR^**^	0.9614	0.9671	0.9585	0.9131	0.9063
		WS	0.9758	0.9693	0.9709	0.9385	0.9717
		NMI	0.9675	0.9678	0.9479	0.9415	0.8923
					
GM12878	50/100	OR	0.9174	0.8136	0.9328	0.8639	0.8546
		WS	0.9374	0.8866	0.9545	0.9068	0.9574
		NMI	0.9398	0.9248	0.9396	0.9343	0.8748
					
IMR90	25/50	OR	0.9042	0.7990	0.9106	0.7004	0.8108
		WS	0.9343	0.8784	0.9457	0.8226	0.8670
		NMI	0.9412	0.9296	0.9407	0.9078	0.7598
					
IMR90	50/100	OR	0.9069	0.8995	0.8990	0.9181	0.8861
		WS	0.9320	0.9310	0.9377	0.9377	0.9556
		NMI	0.9461	0.9410	0.9171	0.9410	0.9072

* 25/50: agreement of 25 kb v.s. 50 kb; 50/100: agreement of 50 kb v.s. 100 kb

^**^ OR=overlapping ratio; WS=weighted similarity

### 3.6 Experiments on simulated unordered graph

**Fig. 8:**
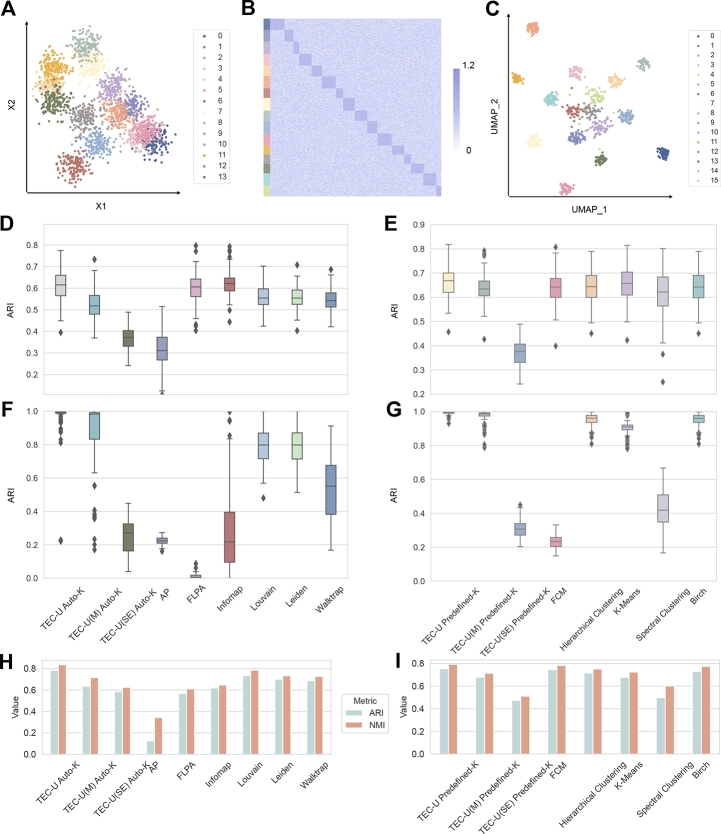
Performance comparison of methods on two types of simulated datasets. A. A two-dimensional input matrix. B. A higher-dimensional input matrix. C. UMAP dimensionality reduction of the matrix from B. The colors of the points in A and C, along with the colored bars on the left side of B, indicate the different clusters obtained by TEC-U. D-G. The ARI distributions of the methods under predefined-*K* and auto-*K* modes, respectively, after conducting 100 repeated experiments on the two-dimensional data in D-E and the higher-dimensional data in F-G. H-I. The averaged ARI and NMI values of the methods under auto-*K* and predefined-*K* modes, respectively, after 100 repeated experiments on the simulated single-cell data.

We generated two types of input data: two-dimensional and higher-dimensional datasets, to evaluate the algorithmic framework of TEC-U and assess topology entropy as an objective measure. Specifically, the higher-dimensional dataset refers to high-dimensional matrices processed into cell-cell similarity graphs. Examples of these two types of input data are shown in Fig. 8A and B.

For the two-dimensional data, we generated isotropic Gaussian blobs for clustering. We fixed the number of clusters at 14, with each cluster containing 150 members and a standard deviation of 1.2. In contrast, the higher-dimensional data were simulated under different settings to mimic single-cell sequencing data. The simulated input matrix was initialized with randomly generated values ranging from 0 to 1. For each cell cluster, we designated approximately 20 marker genes (ranging from 8 to 32 across our experiments). Specifically, for cells in cluster *C*, certain genes exhibited expression values that were moderately higher than those of cells outside the cluster. Our goal was to compare methods that leverage features represented by these marker genes. To increase complexity, some expression values within the cluster were allowed to be lower than those outside, with the range set from 0.5 to 1.2. The higher-dimensional data comprised 16 clusters, each containing between 40 and 60 members (or cells). The simulation data were generated independently 100 times under each setting and subsequently input into all methods.

**Fig. J.14:**
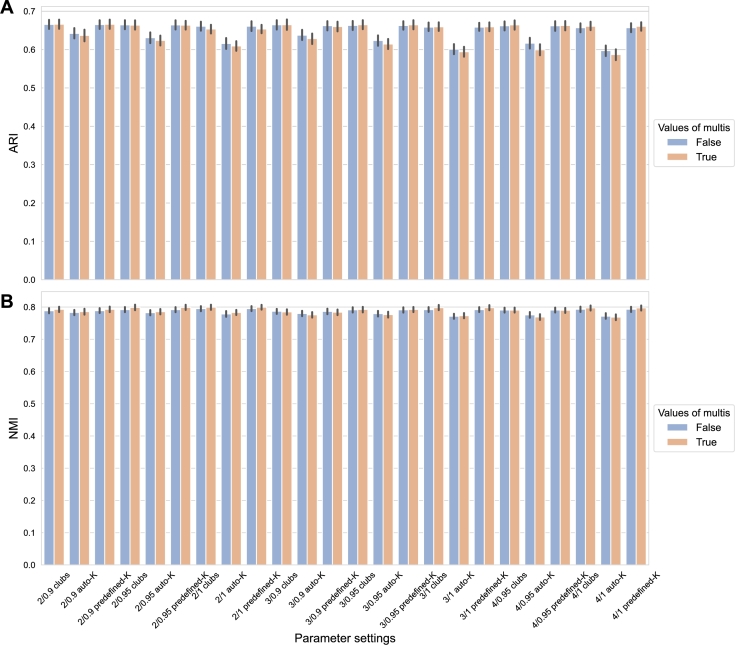
Comparison of different *multis* settings in the TEC-U algorithm under various (*α*_1_/*α*_2_) configurations. Here, *multis* is a boolean variable with possible values of True and False. The term *clubs* represents the results of only running the merging phase of the TEC-U algorithm without executing the combination phase. The *predefined-K* and *auto-K* settings correspond to the two modes provided in TEC-U, where predefined-*K* uses a given *K* value and auto-K automatically determines the optimal *K*.

For comparison, we calculated the Adjusted Rand Index (ARI) [33] with respect to the ground truth to measure the concordance (details in Appendix E). ARI values range from -1 to 1, where an ideal clustering achieves an ARI value of 1, and a value close to 0 indicates random clustering. For details on parameter settings, please see Fig. J.14.

We compared the results from several clustering algorithms, including Louvain, Leiden, Walktrap [34], FLPA [35], Affinity Propagation (AP) [36], and Infomap for the auto-*K* mode; Fuzzy C-Means (FCM) [37], Hierarchical Clustering, K-Means, Spectral Clustering, and Birch [38] for the predefined-*K* mode. For predefined-*K* mode, *K* was provided with the ground truth value. To validate the effectiveness of our proposed topology entropy and the overall performance of our algorithmic framework, we substituted the topology entropy in TEC-U with modularity and structural entropy, resulting in TEC-U(M) and TEC-U(SE), respectively. All baseline methods utilized their recommended or default parameters, requiring input in the form of a kNN graph or other specified formats; thus, data were processed accordingly. The value of $\Delta D_{thres}$ in the merging phase of TEC-U was set to 0.0000001.

**Fig. K.15:**
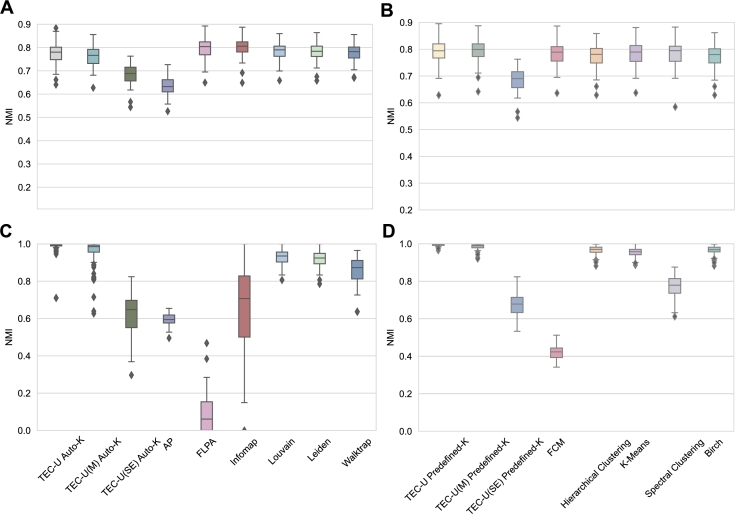
Performance comparison of methods on two types of simulated datasets. A-B. The NMI distributions of the methods under predefined-*K* and auto-*K* modes, respectively, after conducting 100 repeated experiments on the two-dimensional data. C-D. The NMI distributions of the methods under predefined-*K* and auto-*K* modes, respectively, after 100 repeated experiments on the *M*-dimensional data.

As shown in Fig. 8, in auto-*K* mode, Louvain, Leiden, and TEC-U achieved higher average ARI scores on higher-dimensional data, while FLPA and Infomap demonstrated better performance on the two-dimensional data. In predefined-*K* mode, for two-dimensional data, the performance of all methods was relatively similar, with the exception of the one using structural entropy, with TEC-U performing slightly better than K-Means. For higher-dimensional data, FCM and Spectral Clustering showed a significant decline in performance, while Hierarchical Clustering, K-Means, Birch, and TEC-U demonstrated higher accuracy. For higher-dimensional data, the median ARI of TEC-U was higher than that of other methods in both auto-*K* and predefined-*K* modes. Even when using the same objective function, modularity, TEC-U(M) achieved a higher median ARI compared to Louvain and Leiden, validating the efficiency of TEC-U's design. Additionally, the comparison indicates that under the same algorithmic framework, the definition of topology entropy can better facilitate accurate clustering. Results of NMI scores are provided in Fig. K.15.

To further evaluate the performance on single-cell clustering, simulated single-cell data were generated using splatter [39], using the following parameters: three cell populations, nGenes=2,000, group.prob = c(0.4, 0.3,0.3), and path.from=c(0,0,1). The sizes of the datasets were 100, 300, 500, 1000, 2000, 5000, and 10,000, respectively. Simulated data of each size are generated randomly 100 times. The ground truth cell populations were obtained during generation. As shown in Fig. 8H and I, TEC-U achieved higher accuracy in both modes (further details in Table K.13, Table K.14). Additionally, the performance of TEC-U was compared with results obtained without utilizing sparse and dense graphs generated during the graph construction process. The results presented in Table K.15, Table K.16 demonstrate the effectiveness of graph construction in TEC-U.

### 3.7 Determining cell hierarchy on scRNA and scATAC data

For experiments, we collected eight scRNA-seq datasets and two scATAC-seq datasets. The scRNA-seq datasets include those from Yan [40], Deng [41], Biase [42], Goolam [43], Koh [44], Kumar [45], Trapnell [46], and Xin [47]. We downloaded the expression profiles and corresponding cell labels for the scRNA-seq data (accession codes are summarized in Appendix G), and performed dimensionality reduction using UMAP [48]. For the scATAC-seq datasets, namely scatac_6cl [49] and T-cell [50], we utilized processed accessibility profiles and cell labels from scOpen [51], which were also reduced in dimensionality using UMAP. Regarding the TEC-U algorithm, we recommend processing the raw data to obtain low-dimensional embeddings as input.

**Fig. 9:**
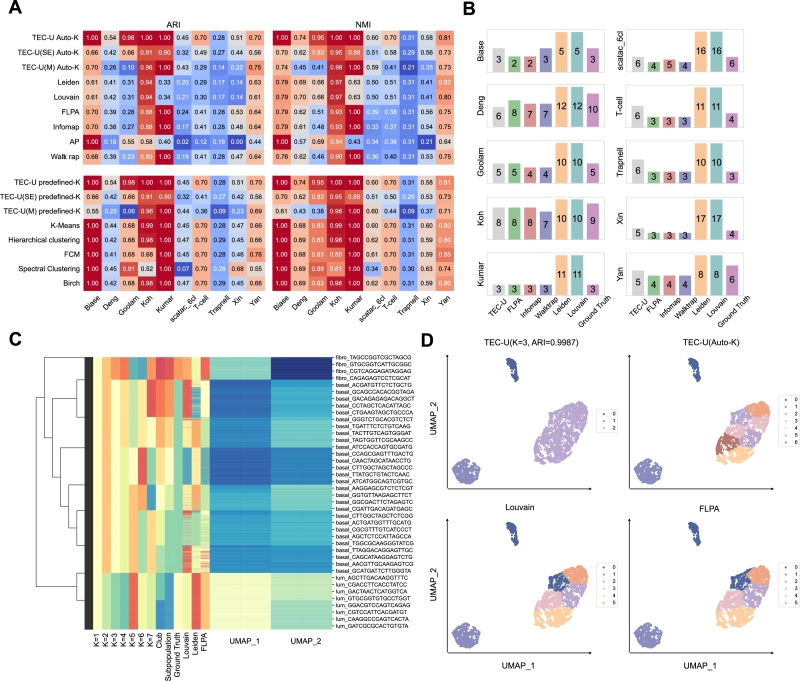
Applying methods to scRNA and scATAC datasets. A. The ARI and NMI scores of the methods in auto-*K* and predefined-*K* modes for each dataset. Darker red indicates higher metric values, while darker blue indicates lower values. B. The number of subpopulations detected by the methods for each dataset in auto-*K* mode. C. The clustering results on the p3cl dataset of TEC-U corresponding to different predefined *K* values. UMAP dimensionality reduction was performed on the p3cl data. The term *club* refers to the result of only conducting the merging phase in TEC-U. For comparison, the ground truth and the clustering results of Louvain, Leiden, and FLPA are also included. D. UMAP plots of the p3cl dataset, with cells colored by clusters. The ARI score of TEC-U is 0.9987.

To evaluate the performance of the methods in cell subpopulation detection, we used ARI and NMI as metrics for clustering accuracy. Comparisons were conducted for both predefined-*K* and auto-*K* modes. As illustrated in Fig. 9A, TEC-U, utilizing topology entropy, outperforms or matches the accuracy of other methods across most datasets, except for Xin and Yan. Even in the auto-*K* mode, our algorithm framework, which employs modularity and structural entropy as the objective function, demonstrates high accuracy. Additionally, it is evident that TEC-U exhibits overall stability in accuracy, without the significant discrepancies between maximum and minimum accuracy observed in methods such as AP and Spectral Clustering. Louvain and Leiden show very similar performance across all datasets. In Fig. 9B, it is evident that across the ten datasets we used, Louvain and Leiden tend to produce far more clusters than the ground truth, resulting in lower clustering accuracy. In contrast, TEC-U yields *K* values in the auto-*K* mode that are closer to the ground truth.

To further observe and compare the results of cell subpopulation detection, we applied methods to the p3cl dataset [52], which includes three cell lines: human dermal fibroblasts, the breast cancer luminal epithelial cell line MCF-7, and the breast cancer basal-like epithelial cell line MDA-MB-468, all obtained through 10x single-cell sequencing, comprising a total of 2,609 cells. As shown in Fig. 9C-D, the clustering result of TEC-U is closer to the ground truth and can simultaneously provide finer resolution cell clusters based on subpopulations, thereby constructing a cell hierarchy.

## 4 Conclusion

In this study, we introduce topology entropy, which quantifies the complexity of networks. To facilitate graph partitioning for both ordered and unordered biological graphs, we developed the encoding tree with minimal topology entropy. For ordered graphs, we present an optimal algorithm named TEC-O, which leverages DP and operates in polynomial time. For unordered graphs, we design a heuristic algorithm called TEC-U, which constructs the topology entropy encoding tree in an agglomerative manner and employs DP to contract the hierarchical tree, yielding the corresponding partitioning results. Additionally, TEC-U offers two operational modes, depending on whether the value of *K* is specified in advance. We validate our approaches using both simulated and real datasets. TEC-O and TEC-U are tested across various noise ratios and cluster sizes on simulated data. Performance comparisons demonstrate that our topology entropy metric effectively encodes structural information, providing a robust foundation for knowledge discovery from noisy data. Experiments on real Hi-C and single-cell sequencing data demonstrate that our methods outperform other baseline approaches and yield biologically meaningful results.

In future research, we aim to provide a more comprehensive theoretical analysis of the definition of topology entropy and investigate its accessibility and robustness as a metric. Furthermore, we plan to explore additional applications in biological problems to expand the utility of our proposed methods.

## CRediT authorship contribution statement

**Qiushi Liang:** Writing – review & editing, Writing – original draft, Validation, Software, Methodology, Investigation, Formal analysis. **Shengjie Zhao:** Writing – review & editing, Project administration, Funding acquisition. **Lingxi Chen:** Writing – review & editing, Investigation. **Shuai Cheng Li:** Writing – review & editing, Project administration, Methodology, Conceptualization.

## Declaration of Competing Interest

None.

## Figures and Tables

**Fig. A.10:**
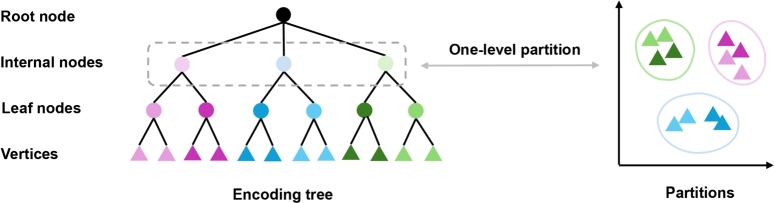
The correspondence between the encoding tree and one-level (or one-layer) partitions.

**Algorithm 1:**
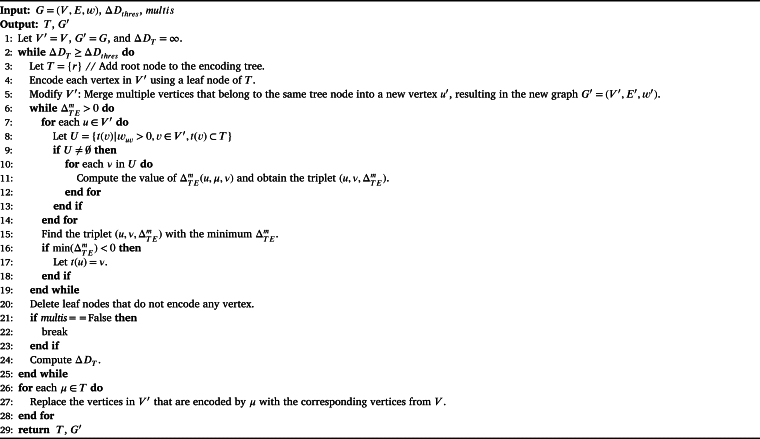
Algorithm for merging phase in TEC-U.

**Table F.2:** Fold change of regulatory elements on chromosome 19 for K562 cell line, within TADs comprising two levels.

Method	CTCF	H3K27ac	H3K4me3	H3K9me3	RAD21	SMC3
TEC-O	0.6703	0.2261	0.5803	-0.4676	0.7406	0.4451
TEC-O(M)	0.3734	0.0969	0.3282	-0.1712	0.5689	0.4285
SuperTAD	0.4142	0.1866	0.4258	-0.5118	0.7351	0.4418
deDoc2	0.3891	0.1534	0.5079	-0.1463	0.8088	0.3658
3DNetMod	0.4577	0.6148	0.6183	-0.3317	0.6349	0.4000

**Table G.3:** Accession numbers of ChIP-seq data derived on real Hi-C cell lines.

Cell lines	Regulatory elements	Accession number
GM12878	CTCF	ENCFF710VEH
	RAD21	ENCFF753RGL
	SMC3	ENCFF496PLN
	H3K27me3	ENCFF594HSG
	H3K36me3	ENCFF662QFK

IMR90	CTCF	ENCFF453XKM
	RAD21	ENCFF195CYT
	SMC3	ENCFF770ISZ
	H3K27me3	ENCFF062LIE
	H3K36me3	ENCFF039DZS

K562	CTCF	ENCFF002DDJ
	H3K27ac	ENCFF044JNJ
	H3K4me3	ENCFF378OQB
	H3K9me3	ENCFF894VEM
	RAD21	ENCFF002CXU
	SMC3	ENCFF041YQC

NHEK	CTCF	ENCFF226MQR
	H3K27ac	ENCFF943CBQ
	H3K4me3	ENCFF027FJC
	H3K9me3	ENCFF188QAF

HMEC	CTCF	ENCFF288RFS
	H3K27ac	ENCFF360PVA
	H3K4me3	ENCFF737CON
	H3K9me3	ENCFF979VXO

Micro-C human	CTCF	ENCFF023LAA
embryonic stem cells	H3K4me3	ENCFF277AOQ
	H3K9me3	ENCFF678VNN
	RAD21	ENCFF255FRL

**Table G.4:** GEO accession numbers of scRNA-seq datasets.

Dataset	Accession number
Biase [42]	GSE57249
Deng [41]	GSE57249
Goolam [43]	E-MTAB-3321
Xin [47]	GSE81608
Yan [40]	GSE45719

**Table H.5:** Fold change of structural proteins peak number and p-value on chromosome 19 for cell line GM12878.

Method	CTCF		RAD21		SMC3	
	Value	pvalue	Value	pvalue	Value	pvalue
3DNetMod	0.3622	1.476597e-14	0.209677	0.000218	0.221769	0.122002
SuperTAD	0.966667	5.247207e-04	0.890909	0.004407	0.769231	0.043140
TEC-O(M)	0.942857	1.682791e-03	0.705882	0.030459	1.000000	0.022750
deDoc2	1.192308	1.036180e-04	0.918367	0.001632	0.615385	0.040449
TEC-O	1.211952	1.209546e-05	1.048569	0.001257	1.086957	0.003511

**Table H.6:** Fold change of structural proteins peak number and p-value on chromosome 19 for cell line IMR90.

Method	CTCF		RAD21		SMC3	
	Value	pvalue	Value	pvalue	Value	pvalue
TEC-O	1.048104	1.443290e-15	0.887897	0.000010	1.181818	5.927131e-03
TEC-O(M)	0.888889	2.377124e-02	0.933333	3.242684e-02	1.285714	3.343046e-03
SuperTAD	1.034483	3.986831e-07	1.000000	0.000090	1.555556	1.055913e-02
deDoc2	0.932203	2.980990e-05	0.843137	0.000077	1.333333	7.344163e-03
3DNetMod	0.631563	7.449641e-07	0.648352	0.000080	0.607741	1.670864e-05

**Table H.7:** Fraction of TADs from three groups on chromosome 19 for cell line GM12878: enriched for H3K27me3, H3K36me3, and no significant enrichment.

Method	H3K27me3	H3K36me3	FDR>0.1
TEC-O	0.3889	0.5556	0.0556
TEC-O(M)	0.5000	0.4167	0.0833
SuperTAD	0.3810	0.4762	0.1429
deDoc2	0.4286	0.4762	0.0952
3DNetMod	0.4149	0.4362	0.1489

**Table H.8:** Fraction of TADs from three groups on chromosome 19 for cell line IMR90: enriched for H3K27me3, H3K36me3, and no significant enrichment.

Method	H3K27me3	H3K36me3	FDR>0.1
TEC-O	0.4667	0.4667	0.0667
TEC-O(M)	0.4667	0.4667	0.0667
SuperTAD	0.4000	0.4667	0.1333
deDoc2	0.4688	0.4375	0.0938
3DNetMod	0.3291	0.4684	0.2025

**Table I.9:** Fold change of regulatory elements on chromosome 19 for K562 cell line.

Method	CTCF	H3K27ac	H3K4me3	H3K9me3	RAD21	SMC3
TEC-O	0.4510	1.0430	0.6621	-0.6667	1.3077	0.5758
TEC-O(M)	0.5331	0.3472	0.6207	-0.4211	1.0000	0.5200
SuperTAD	0.4054	0.3975	0.5674	-0.7576	0.6111	0.2632
deDoc2	0.3333	0.4087	0.5110	-0.7241	0.4359	0.2533
3DNetMod	0.4577	0.6148	0.6183	-0.3317	0.6349	0.4000

**Table I.10:** Fold change of regulatory elements on chromosome 19 for NHEK and HMEC cell lines.

Cell line	Regulatory Element	TEC-O	TEC-O(M)	SuperTAD	deDoc2	3DNetMod
NHEK	CTCF	1.0000	0.8427	0.6814	0.7593	0.8068
	H3K27ac	1.1765	0.5566	0.3530	0.3289	1.1459
	H3K4me3	0.9968	0.8046	0.4841	0.5709	1.0599
	H3K9me3	-0.4166	-0.4138	-0.4430	-0.4286	-0.6005

HMEC	CTCF	0.2709	0.3383	0.2754	0.3145	0.4067
	H3K27ac	0.4539	0.3414	0.2450	0.2769	0.4016
	H3K4me3	0.7785	0.7076	0.3438	0.3590	0.5955
	H3K9me3	-0.4227	-0.6147	-0.2213	-0.5482	-0.6277

**Table I.11:** TAD inference performance via TAD callers across multiple ratios of downsampled sequenced reads.

Ratio of downsampled	Statistics	Method				
		TEC-O	TEC-O(M)	SuperTAD	deDoc2	3DNetMod
30%	Number of TADs	28	16	23	23	4
	Average TAD size (Mb)	0.4857	0.8875	0.6021	0.6021	0.1375
	OR	0.9983	0.7083	0.9900	0.9850	0.0148
	WS	0.9983	0.7798	0.9903	0.9862	0.4186

50%	Number of TADs	28	16	23	23	3
	Average TAD size (Mb)	0.4857	0.8890	0.6021	0.6021	0.1416
	OR	0.9983	0.78	0.9917	0.9883	0.011
	WS	0.9985	0.8268	0.9920	0.9895	0.4107

70%	Number of TADs	28	16	23	24	4
	Average TAD size (Mb)	0.4857	0.8875	0.6021	0.5760	0.125
	OR	1	0.7050	0.9945	1	0.0103
	WS	1	0.7810	0.9987	1	0.3188

**Table I.12:** Fold change of regulatory elements over normalized interaction dataset of Micro-C human embryonic stem cell line on chromosome 19 at 5 kb and 10 kb resolutions. The evaluation of 3DNetMod is omitted because only a small part of bins are partitioned.

Resolution	Method	CTCF	H3K4me3	H3K9me3	RAD21
5 kb	TEC-O	2.6000	3.2699	0.4118	3.2500
	TEC-O(M)	1.8165	1.5004	-0.0107	1.5543
	SuperTAD	1.4969	1.7833	0.3161	1.2650
	deDoc2	1.4212	1.7833	0.1979	1.1895

10 kb	TEC-O	0.8000	2.6453	0.0931	0.7333
	TEC-O(M)	0.6364	2.4608	0.2458	0.8000
	SuperTAD	0.7778	1.9001	0.0441	0.6471
	deDoc2	0.8235	1.9001	-0.0621	0.6875

**Table K.13:** Averaged ARI and NMI for different auto-*K* methods on simulated single-cell data.

# of cells	Metric	Methods								
		TEC-U Auto-*K*	TEC-U(M) Auto-*K*	TEC-U(SE) Auto-*K*	AP	FLPA	Infomap	Louvain	Leiden	Walktrap
100	ARI	0.8179	0.6953	0.6866	0.5816	0.6714	0.6198	0.8016	0.7097	0.7210
	NMI	0.8676	0.7599	0.7313	0.6950	0.7001	0.6452	0.8471	0.7261	0.7489

300	ARI	0.8710	0.7313	0.7220	0.2738	0.6719	0.6802	0.8345	0.7474	0.7582
	NMI	0.9137	0.7883	0.7574	0.4303	0.7018	0.6909	0.8752	0.7625	0.7857

500	ARI	0.8150	0.6645	0.6397	0.1499	0.6956	0.6681	0.7780	0.7421	0.7178
	NMI	0.8623	0.7346	0.6873	0.3412	0.7247	0.6821	0.8258	0.7645	0.7484

1000	ARI	0.8151	0.6667	0.6374	0.1179	0.5850	0.6361	0.7929	0.7158	0.6944
	NMI	0.8695	0.7411	0.6731	0.3052	0.6326	0.6524	0.8410	0.7420	0.7310

2000	ARI	0.7639	0.6014	0.5102	0.0532	0.5047	0.5799	0.7100	0.6697	0.6718
	NMI	0.8222	0.6924	0.5579	0.2574	0.5702	0.6089	0.7637	0.7136	0.7237

5000	ARI	0.6974	0.5544	0.4963	0.0343	0.4358	0.5723	0.6655	0.6552	0.6086
	NMI	0.7593	0.6530	0.5317	0.2383	0.4841	0.6158	0.7288	0.7021	0.6597

10000	ARI	0.7749	0.5925	0.5077	0.0405	0.5459	0.6216	0.6251	0.7091	0.6982
	NMI	0.8148	0.6840	0.5422	0.2359	0.5846	0.6576	0.6801	0.7469	0.7418

**Table K.14:** Averaged ARI and NMI for different predefined-*K* methods on simulated single-cell data.

# of cells	Metric	Methods							
		TEC-U Predefined-*K*	TEC-U(M) Predefined-*K*	TEC-U(SE) Predefined-*K*	FCM	Hierarchical Clustering	K-Means	Spectral Clustering	Birch
100	ARI	0.7991	0.6867	0.5734	0.8042	0.7279	0.8205	0.7453	0.8075
	NMI	0.8297	0.7198	0.6218	0.8393	0.7510	0.8522	0.8006	0.8366

300	ARI	0.8491	0.7577	0.6340	0.7971	0.7759	0.8132	0.7047	0.8118
	NMI	0.8778	0.7764	0.6702	0.8311	0.7936	0.8485	0.7745	0.8451

500	ARI	0.7896	0.7173	0.5163	0.7909	0.7425	0.8032	0.6029	0.7722
	NMI	0.8267	0.7404	0.5695	0.8251	0.7679	0.8357	0.6952	0.8070

1000	ARI	0.7640	0.7018	0.5229	0.7602	0.7375	0.7592	0.5845	0.7423
	NMI	0.8135	0.7348	0.5634	0.7923	0.7645	0.7999	0.6703	0.7842

2000	ARI	0.7651	0.6224	0.3996	0.6695	0.7167	0.7017	0.3682	0.6557
	NMI	0.8046	0.6672	0.4372	0.7146	0.7512	0.7478	0.4835	0.7151

5000	ARI	0.6764	0.5949	0.4025	0.6857	0.6301	0.7116	0.3286	0.6468
	NMI	0.7261	0.6416	0.4262	0.7274	0.6841	0.7486	0.4528	0.6999

10000	ARI	0.6738	0.6814	0.3695	0.7787	0.7690	0.7652	0.3289	0.7436
	NMI	0.7105	0.7294	0.4016	0.8111	0.8146	0.7980	0.4863	0.7934

**Table K.15:** Averaged ARI and NMI for TEC-U with different graph construction methods (auto-*K* mode). ‘PCC’ and ‘cosine similarity’ refer to the use of PCC or cosine similarity to compute the similarity graph.

# of cells	Metric	Graph construction			
		only sparse	only dense	PCC	cosine similarity
100	ARI	0.5924	0.1519	0.3882	0.5463
	NMI	0.6541	0.4555	0.4312	0.5804

300	ARI	0.6819	0.0604	0.4834	0.4834
	NMI	0.7143	0.3602	0.5398	0.5398

500	ARI	0.7160	0.0000	0.5495	0.5479
	NMI	0.7334	0.0000	0.5864	0.5846

1000	ARI	0.5346	0.0000	0.5462	0.5420
	NMI	0.6290	0.0000	0.5683	0.5538

2000	ARI	0.4210	0.0022	0.3288	0.3297
	NMI	0.5164	0.0318	0.4157	0.4175

5000	ARI	0.5801	0.0027	0.5248	0.5246
	NMI	0.6693	0.0039	0.5682	0.5680

10000	ARI	0.6092	0.0018	0.5167	0.5157
	NMI	0.6688	0.0033	0.5622	0.5613

**Table K.16:** Averaged ARI and NMI for TEC-U with different graph construction methods (predefined-*K* mode). ‘PCC’ and ‘cosine similarity’ refer to the use of PCC or cosine similarity to compute the similarity graph.

# of cells	Metric	Graph construction			
		only sparse	only dense	PCC	cosine similarity
100	ARI	0.4344	0.0217	0.3882	0.5463
	NMI	0.5449	0.2535	0.4312	0.5804

300	ARI	0.3583	0.0671	0.4834	0.4834
	NMI	0.4610	0.3602	0.5398	0.5398

500	ARI	0.3771	0.0000	0.5495	0.5479
	NMI	0.4757	0.2976	0.5864	0.5846

1000	ARI	0.3006	0.0000	0.5421	0.5420
	NMI	0.4030	0.0000	0.5532	0.5561

2000	ARI	0.4236	0.0015	0.3288	0.3297
	NMI	0.5183	0.0077	0.4157	0.4175

5000	ARI	0.5478	0.0000	0.5248	0.5246
	NMI	0.6475	0.0000	0.5682	0.5680

10000	ARI	0.3383	0.0006	0.5167	0.5157
	NMI	0.4313	0.0013	0.5622	0.5613

## Data Availability

All data are publicly available. The details are summarized in Results.

## Appendix A Definition of modularity

The original modularity definition in [28] is

(A.1) $$M(G)=\frac{1}{2m}\sum_{u,v}\left[A_{uv}-\frac{k_{u}k_{v}}{2m}\right]\delta (c_{u},c_{v}),$$

where $A_{uv}$ is an element of the 0-1 adjacency matrix for graph *G*, *u* and *v* represent vertices in *G* and belong to communities $c_{u}$ and $c_{v}$, respectively, $m=\frac{1}{2}\sum_{u,v}A_{uv}$ represents the number of edges in the graph, $k_{u}$ is the degree of vertex *u*, and the *δ*-function δ(u,v) is 1 if i=j and 0 otherwise. The term $\frac{k_{u}k_{v}}{2m}$ identifies the probability of an edge occurring between vertices *u* and *v* when connections are randomly established, yet adhering to the degree constraints of the vertices. According to [53], Eq. (A.1) can be interpreted as the number of edges within groups, as well as the difference between that number and the expected number of such edges, assuming edges are placed at random within the network. If the proportion of edges within the community matches the expected value for the randomized network, then this measure will be zero [54].

To address the issue of the limited scale of communities, Arenas et al. [28] proposed a generalized modularity function that can be written in the form

(A.2) $$M(G)=\frac{1}{2m}\sum_{uv}\left[A_{uv}-\gamma \frac{k_{u}k_{v}}{2m}\right]\delta (c_{u},c_{v}).$$

Based on Eq. (A.2), we have

(A.3) $$M(G)=\sum_{C}\sum_{u,v\in C}\left[\frac{A_{uv}}{2m}-\gamma \frac{k_{u}k_{v}}{{\left(2m\right)}^{2}}\right].$$

At this point, the communities correspond to a non-hierarchical partition. We now aim to compute the modularity of the hierarchical encoding tree. The correspondence between the encoding tree and one-level (or one-layer) partitions is illustrated in Fig. A.10. According to the definition of the encoding tree (Section 2.1.1), we have vol(G)=2m, $\sum_{u,v\in C}A_{uv}=s(C)$, and $\sum_{u\in C}k_{u}\sum_{v\in C}k_{v}=vol^{2}(C)$. Therefore, for a one-layer partition, we have

(A.4) $$M(G)=\sum_{C}\left[\frac{s(C)}{vol(G)}-\gamma {\left(\frac{vol(C)}{vol(G)}\right)}^{2}\right].$$

The tree node *μ* that we defined is equivalent to community *C*. We define the total modularity of the encoding tree as the sum of the modularity corresponding to all layers. Therefore, for the encoding tree *T*, we have

(A.5) $$M_{T}(G)=\sum_{\mu \in T}\left[\frac{s(\mu )}{vol(G)}-\gamma {\left(\frac{vol(\mu )}{vol(G)}\right)}^{2}\right].$$

Clearly, the modularity of node *μ* is as Eq. (1) in Section 2.1.2.

The modularity of vertex *u* is

(A.6) $$M_{T}(G;u)=-\gamma {\left(\frac{vol(u)}{vol(G)}\right)}^{2}.$$

The modularity of vertices encoded by node t(u) is

(A.7) $$M_{T}(G;V(t(u)))=\sum_{u\in V(t(u))}-\gamma {\left(\frac{vol(u)}{vol(G)}\right)}^{2}=-\frac{\gamma }{vol{\left(G\right)}^{2}}\sum_{u}{\left(vol(u)\right)}^{2}.$$

## Appendix B From KL divergence to topology entropy

We substitute *P* and *Q* from Eq. (2) into Eq. (3), yielding:

(B.1) $$D(G)=\sum_{\mu }\frac{s(\mu )}{vol(G)}\left(\log\frac{s(\mu )}{vol(G)}-\log\gamma -2log\frac{vol(\mu )}{vol(G)}\right).$$

Based on Eq. (B.1), we obtain the expression for each tree node *μ* as

(B.2) $$D(G;\mu )=\frac{s(\mu )}{vol(G)}\left(\log\frac{s(\mu )}{vol(G)}-\log\gamma -2log\frac{vol(\mu )}{vol(G)}\right).$$

The term log⁡γ represents a constant; hence, we omit this term. To address the issue of the limited scale of communities, we introduce parameters $\alpha_{1}$ and $\alpha_{2}$ into the definition, similar to *γ* in Eq. (1):

(B.3) $$D(G;\mu )=-{\left(\frac{s(\mu )}{vol(G)}\right)}^{\alpha_{1}}\left(\log\alpha_{1}\frac{s(\mu )}{vol(G)}-\alpha_{2}\log\frac{vol(\mu )}{vol(G)}\right).$$

Since the two terms within the parentheses can be controlled by a single parameter and their weights are related, we remove the addition of $\alpha_{1}$ from the second $\log\frac{s(\mu )}{vol(G)}$ in Eq. (B.3) and obtain Eq. (4) in Section 2.1.2.

## Appendix C Structural entropy and topology entropy

Denote the parent of a tree node *μ* as p(μ). Given a node μ∈T, the structural entropy of it is

(C.1) $$S_{T}(G;\mu )=-\frac{g(\mu )}{vol(G)}\log_{2}\frac{vol(\mu )}{vol(p(\mu ))},$$

where $S_{T}(G;r)=0$. The optimal structural entropy encoding tree problem is to construct a tree $T_{\text{opt}}^{s}(G)$ that minimizes the structural entropy for graph *G*.

According to Eq. (4), let $\alpha_{1}=1$, the topology entropy can be written as

(C.2) $$D(G;\mu )=-\frac{s(\mu )}{vol(G)}\log\frac{s(\mu )}{vol(G)}+\alpha_{2}\frac{vol(\mu )}{vol(G)}\log\frac{vol(\mu )}{vol(G)}-\alpha_{2}\frac{g(\mu )}{vol(G)}\log\frac{vol(\mu )}{vol(G)}.$$

As s(μ)→v(μ) and $\alpha_{2}=1$, we have $D(G;\mu )\to S_{T}(G;\mu )$. In other words, the optimal structures obtained using these two entropy values as metrics can be similar.

## Appendix E Metrics for assessing the similarity of encoding trees

Let *X* and *Y* denote the ground truth structure and the inferred structure, respectively, for the same input matrix. Specifically, $X=\left\{x_{1},\ldots ,x_{m}\right\}$ and $Y=\left\{y_{1},\ldots ,y_{n}\right\}$ represent the sets of tree nodes, excluding the root node, with |X|=m, and |Y|=n. Each node in *X* or *Y* corresponds to a consecutive set of bins.

The weighted similarity [7] between *X* and *Y* is defined as

(E.1) $$ws_{X}^{Y}=\frac{\sum_{i=1}^{n}|y_{i}|\cdot S_{X}^{Y}(i)}{\sum_{i=1}^{n}y_{i}},$$

where

(E.2) $$S_{X}^{Y}(i)=\max\left\{\frac{\left|x_{j}\cap y_{i}\right|}{\sqrt{\left|x_{j}|\cdot |y_{i}\right|}}\right\}.$$

Note that the weighted similarity is asymmetric.

We construct a bipartite graph where each edge connects a node in *X* to a node in *Y*. Let *M* denote the maximum matching of this graph, obtained using maximum bipartite matching. The overlapping ratio [9] between *X* and *Y* is defined as

(E.3) $$or(X,Y)=\frac{\sum_{i=1}^{m}w^{\prime }(x_{i},⁎)+\sum_{j=1}^{n}w^{\prime }(⁎,y_{j})}{\sum_{i=1}^{m}|x_{i}|+\sum_{j=1}^{n}|y_{j}|},$$

where $w^{\prime }$ represents the weight of edges in *M*, defined as

(E.4) $$w^{\prime }(x_{i},⁎)\left\{ \begin{array}{cc} w(x_{i},y_{j}), & \text{if edge }e(x_{i},y_{j})\text{ is in }M\\ 0, & \text{otherwise} \end{array}\right.$$

The overlapping ratio is symmetric.

The Adjusted Rand Index (ARI) [33] is defined as

(E.5) $$ARI=\frac{RI-E(RI)}{\max(RI)-E(RI)},$$

where *RI* denotes the Rand Index, defined as

(E.6) $$RI=\frac{a+b}{\left(\begin{array}{c} n\\ 2 \end{array}\right)},$$

with *a* representing the number of vertices that are in the same cluster in both *X* and *Y*, and *b* representing the number of vertices that are in the different clusters in *X* but in the same cluster in *Y*. The ARI is a symmetric measure.

The Normalized Mutual Information (NMI) is defined as

(E.7) $$NMI(X,Y)=\frac{2⁎I(X;Y)}{H(X)+H(Y)},$$

where H(⋅) denotes the entropy, and I(X;Y) is the mutual information, defined as

(E.8) I(X;Y)=H(X)−H(X|Y).

The NMI is symmetric and measures the agreement of partitions while ignoring permutations.

## Appendix G Accession numbers of dataset used in experiments

The TPM expression of HGV10 genes and cell labels of datasets Koh, Kumar, and Trapnell are obtained from [23]. Furthermore, the raw count expression profiles and cell type labels for the p3cl dataset are downloaded with GEO accession code GSE136148.

## Appendix K Comparison of methods using simulated datasets for unordered graphs

To validate the performance of the graph construction in TEC-U, we conducted experiments using only a sparse graph (denoted as ‘only sparse’), only a dense graph (denoted as ‘only dense’), and graphs constructed using the Pearson Correlation Coefficient (PCC) and cosine similarity on the simulated single-cell dataset. As shown in Table K.15, Table K.16, all configurations exhibited lower accuracy compared to our proposed graph construction method, demonstrating the effectiveness of combining dense and sparse graph settings.

It is important to note that in subsequent experiments, we made every effort to minimize the influence of graph construction on the results. Specifically, for baseline methods requiring a sparse graph, we used the kNN graph generated during the graph construction process. Conversely, for methods requiring a dense graph, we utilized the dense graph produced using the Gaussian kernel.
